# Supplementary material for: HIVprotI: an integrated web based platform for prediction and design of HIV proteins inhibitors
Source: J Cheminform. 2018 Mar 9;10:12. doi: 10.1186/s13321-018-0266-y (PMC5845081; doi:10.1186/s13321-018-0266-y)
Supplement: Supplementary file 2 — Additional file 2. Source code of HIVProtI web server. [file 13321_2018_266_MOESM2_ESM.zip › HIVprotI_Source-code/hivprotI/weka-3-6-6/doc/weka/gui/boundaryvisualizer/DataGenerator.html]

DataGenerator


---


|  |  |  |
| --- | --- | --- |
| **Overview** \| **Package** \| **Class** \| **Tree** \| **Deprecated** \| **Index** \| **Help** \| **Weka's home** | |  |
| **PREV CLASS**   **NEXT CLASS** | **FRAMES**    **NO FRAMES**     **All Classes** |  |
| SUMMARY: NESTED \| FIELD \| CONSTR \| METHOD | DETAIL: FIELD \| CONSTR \| METHOD |  |


---


## weka.gui.boundaryvisualizer Interface DataGenerator

**All Known Implementing Classes:**: KDDataGenerator

---

``` public interface DataGenerator ```

Interface to something that can generate new instances based on
a set of input instances

**Since:**
:   1.0

**Version:**
:   $Revision: 1.4 $

**Author:**
:   Mark Hall

---

| **Method Summary** | |
| --- | --- |
| `void` | `buildGenerator(Instances inputInstances)`             Build the data generator |
| `double[][]` | `generateInstances(int[] indices)`             Generate an instance. |
| `int` | `getNumGeneratingModels()`             Returns the number of generating models used by this DataGenerator |
| `double[]` | `getWeights()`             Get weights |
| `void` | `setSeed(int seed)`             Set a seed for random number generation (if needed). |
| `void` | `setWeightingDimensions(boolean[] dimensions)`             Set the dimensions to be used in computing a weight for each instance generated |
| `void` | `setWeightingValues(double[] vals)`             Set the values of the dimensions (chosen via setWeightingDimensions) to be used when computing instance weights |

**Method Detail**

### buildGenerator

```
void buildGenerator(Instances inputInstances)
                    throws java.lang.Exception
```

:   Build the data generator

    :   **Parameters:**: `inputInstances` - Instances to build the generator from **Throws:**: `java.lang.Exception` - if an error occurs

---


### generateInstances

```
double[][] generateInstances(int[] indices)
                             throws java.lang.Exception
```

:   Generate an instance. Should return a new Instance object

    :   **Returns:**: an `Instance` value **Throws:**: `java.lang.Exception` - if an error occurs

---


### getWeights

```
double[] getWeights()
                    throws java.lang.Exception
```

:   Get weights

    :   **Throws:**: `java.lang.Exception`

---


### setWeightingDimensions

```
void setWeightingDimensions(boolean[] dimensions)
```

:   Set the dimensions to be used in computing a weight for
    each instance generated

    :   **Parameters:**: `dimensions` - an array of booleans specifying the dimensions to be used when computing instance weights

---


### setWeightingValues

```
void setWeightingValues(double[] vals)
```

:   Set the values of the dimensions (chosen via setWeightingDimensions)
    to be used when computing instance weights

    :   **Parameters:**: `vals` - a `double[]` value

---


### getNumGeneratingModels

```
int getNumGeneratingModels()
```

:   Returns the number of generating models used by this DataGenerator

    :   **Returns:**: an `int` value

---


### setSeed

```
void setSeed(int seed)
```

:   Set a seed for random number generation (if needed).

    :   **Parameters:**: `seed` - an `int` value


---


|  |  |  |
| --- | --- | --- |
| **Overview** \| **Package** \| **Class** \| **Tree** \| **Deprecated** \| **Index** \| **Help** \| **Weka's home** | |  |
| **PREV CLASS**   **NEXT CLASS** | **FRAMES**    **NO FRAMES**     **All Classes** |  |
| SUMMARY: NESTED \| FIELD \| CONSTR \| METHOD | DETAIL: FIELD \| CONSTR \| METHOD |  |


---
